# Silencing SATB1 with siRNA inhibits the proliferation and invasion of small cell lung cancer cells

**DOI:** 10.1186/1475-2867-13-8

**Published:** 2013-02-05

**Authors:** Bo Huang, Hongli Zhou, Xiaodong Wang, Zhiliang Liu

**Affiliations:** 1 Department of Thoracic Surgery, the First Affiliated Hospital of Liaoning Medical University, No. 2 People street, 121000, Jinzhou, Liaoning, People’s Republic of China; 2 Department of Kidney diseases, the First Affiliated Hospital of Liaoning Medical University, 121000, Jinzhou, Liaoning, People’s Republic of China

**Keywords:** SATB1, Small cell lung cancer, siRNA interfering, Apoptosis

## Abstract

**Background:**

Small cell lung cancer (SCLC) is a special kind of lung cancers, lymph or blood metastasis of SCLC usually occurs in early stage. Studies in breast and colon cancer showed over expression of SATB1 could promote tumor cell growth and inhibit apoptosis. Therefore, we studied the expression of SATB1 in SCLC.

**Methods:**

The level of SATB1 was analyzed in SCLC tissues, metastatic lymphoid nodes and adjacent normal lung tissues by immunohistochemistry. Meanwhile, small interfering SATB1-targeting RNA was constructed and transfected into human SCLC cell line NCI-H446 to evaluate the effects of SATB1-siRNA on cell proliferation, invasion and apoptosis.

**Result:**

SATB1 protein was overexpressed in SCLC tissues and metastasis lymphoid nodes compared with adjacent normal lung tissues. Compared with control group, SATB1-siRNA inhibits the proliferation and invasion of SCLC cells and induces SCLC cells apoptosis statistically (P<0.05) in vitro.

**Conclusion:**

Our results suggest that SATB1 plays an important role in the metastasis of human SCLC cell.

## Introduction

Small cell lung cancer (SCLC) is a special kind of lung cancer, which comprises 15%-20% of all lung cancers. Different from other lung cancers, SCLC exhibits aggressive behavior with rapid growth and spread to distant sites early [1]. Although SCLC is exquisite sensitivity to chemotherapy and radiation, it is more prone to relapse and recurrence. Special AT-rich sequence binding protein 1 (SATB1) is a nuclear matrix attachment region binding protein (MBP) which participates in higher-order chromatin organization and tissue-specific gene expression [2,3]. SATB1 play an important role in differentiation, and expresses at high level in thymocyte, its expression is down-regulated in mature T cells [4,5]. SATB1 has been reported to promote a metastatic phenotype and correlate with poor prognosis in breast cancer [6]. Its expression has also been associated with unfavourable clinicopathological characteristics and poor prognosis in gastric, liver and colorectal cancer and glioma [7-12]. RNA-interference-mediated knockdown in aggressive cancer cells have altered the expression of over 1,000 genes, this technique effectively inhibited cell capacity of proliferation, invasion, tumor growth and metastasis [6]. Zheng et al. also found SATB1 expression in aggressive rather than non-aggressive breast cancer cells [13]. Similarly, transduction of SATB1into non-metastatic cells led to the invasion of tumors in mice [14], whereas silencing SATB1 lead cells to their normal phenotype and prevented tumor growth and metastasis. RNA interference (RNAi) targeting SATB1 has been successfully used in sarcoma [15], leukemia [16] and pancreatic cells [17], few report has been published concerning the effect of small interfering RNA (siRNA) on the SATB1 gene in SCLC cells, in our study, we investigated the expression of SATB 1 and its role in the pathogenesis of SCLC by knocking down SATB1 in NCI-H446 cells.

## Materials and methods

### SCLC specimens

SCLC specimens were obtained from 29 cases of SCLC patients underwent surgery in the First Affiliated Hospital of Liaoning Medical University from January 2009 to December 2012. Neither chemotherapy nor radiotherapy was conducted in these patients. Acquisition and analysis of these operation sample was approved by the ethics committee of hospital, the patient also signed the informed consent.

### Cell line and cell culture

Human SCLC cell line (NCI-H446) (Shanghai Biological Sciences Institute, China) was used to analysis the expression of SATB 1 protein interfered by siRNA. NCI-H446 cells were cultured in RPMI1640 supplemented with 10% fetal bovine serum (FBS; Hyclone), 10 units/L penicillin G and 100 mg/L streptomycin at 37°C in a humidified atmosphere containing 5% CO2.

### Immunohistochemical method

Immunohistochemical analysis was conducted to observe the expression of SATB 1 in SCLC tissues, metastasis lymphoid node tissue and lung cancer adjacent tissue. Briefly, tissues were fixed with 4% paraformaldehyde and embedded with paraffin using standard methods. IHC was carried out using a specific rabbit polyclonal anti-SATB1 antibody (1:250; Jingmei Biotech Corp, Beijing, China) according to the provided instruction of supplier. The brown staining in cytoplasm was defined as the positive signal. The scoring criteria were used as reported previously [18]. Briefly, the staining proportion was classified as 1 (<10%), 2 (10–25%), 3 (26–75%), and 4 (>75%). The staining intensity was graded as 0 for negative, 1 for weak, 2 for moderate, and 3 for strong staining. The staining index was subsequently obtained by multiplication of the proportion and intensity and calculated index was finally assessed by a simplified score (score 0, index 0–1; score 1, index 2–4; score 2, index 6–8; score 3, index 9–12). Samples with staining score of at least one were classified as the positive staining.

### Vector construction

Based on the SATB1 cDNA sequence in Gene Bank, 3 pairs of synthesized oligonucleotide were designed (Dalian Biotechnologies, Dalian, China). The sequences used were as follows: (Si-1) F: 5^′^- GAT CCC CGG ATT TGG AAG AGA GTG TCT TCA AGA GAG ACA CTC TCT TCC AAA TCC TTT TTG GAA A-3^′^; (Si-1) R: 5^′^- AGC TTT TCC AAA AAG GAT TTG GAA GAG AGT GTC TCT CTT GAA GAC ACT CTC TTC CAA ATC CGG G -3^′^; (Si-2) F: 5^′^- GAT CCC CGT CCA CCT TGT CTT CTC TCT TCA AGA GAG AGA GAA GAC AAG GTG GAC TTT TTG GAA A -3^′^ (Si-2) R: 5^′^- AGC TTT TCC AAA AAG TCC ACC TTG TCT TCT CTC TCT CTT GAA GAG AGA AGA CAA GGT GGA CGG G- 3^′^; (Si-N) (control group) F: 5^′^-GAT CCG CGA GAC CTC AGT ATG TTA CCT GTG AAG CCA CAG ATG GGG TAAC ATA CTG AGG TCT CGC TTT TTT G -3^′^. Oligonucleotide was annealed and ligated with pRNAT- U6.1/ Neo-siRNA using T4 DNA ligase. The three constructed recombinant plasmids- SATB1-siRNA-1, SATB1-siRNA-2, and SATB1-siRNA-N were verified by sequencing and restriction endonuclease-digestion.

### RNA interference

NCI-H446 cells were seeded (2 × 10^5^ cells / well) in six-well plates. After incubation for 24 hours, they were transfected by SATB1-siRNA-1, SATB1-siRNA-2, and SATB1-siRNA-N plasmid in serum-free medium by Lipofectamine 2000 regent (Invitrogen, Carlsbad, CA, USA) according to the manufacturer’s instruction.

### Western blot analysis SATB 1 expression level

Cells were washed twice with ice-cold PBS and then treated with lysis buffer for 30 minutes at 4°C. The supernatants were centrifuged at 12000 g 30 minutes at 4°C. Protein samples were electrophoresed on 7.5% SDS-PAGE gels and transferred to PVDF membrane. Nonspecific reactivity was blocked in 5% nonfat dry milk in TBST for 1 hour at room temperature. The membrane was then treated with rabbit anti-human SATB1 antibody (1:250; Jingmei Biotech Corp, Beijing, China) overnight at 4°C, followed by reaction to goat anti-rabbit antibody (1:500; Jingmei Biotech Corp, Beijing, China). Each sample was also probed with β-actin antibody (Sigma-Aldrich Corp.) as a loading control. The intensity of the detected bands was analyzed using Image J program.

### Cell proliferation assay

Cells were treated with siRNA as described above for 48 hours. Then cells (2 × 10^3^ per well) were plated in 96-well plates cultured from 24 to 96 hours, MTT assay was performed to analysis growth of these cells.

### SCLC cells invasion assays

The invasion of SCLC cells was conducted using Matrigel coated 24-well trans-well chamber (Costar). 48 h later, serum-free RPMI-1640 containing 2.0 × 10^5^ cells was introduced into the upper compartment and the lower compartment contained RPMI-1640 supplemented with 15% FCS. Incubated at 37°C for 24 h, the non-invasive cells from the upper surface of the membrane were removed completely with cotton swabs. The invasive cells attached to the lower membrane surface were fixed with 4% paraformaldehyde and stained with hematoxylin and eosin (HE). The number of invasive cells was counted under microscope (200X).

### FACS analysis

After 48 hours, cells were isolated and stained with Propidium Iodide (PI) (BD Biosciences Clontech, Palo Alto, CA) 48 hours after interfered. They were analyzed using FACS according fluorescence of PI. The fraction of apoptotic cells in the siRNA treated population was determined by the super-enhanced DMax method of WinList software (Verity Software House, Topsham, ME).

### Statistical analysis

SPSS 17.0 software was used in statistical analysis. One-way ANOVA was performed for multiple comparisons. The frequencies of MEKK1 expression among cancer samples were analyzed by the test with modification by the Fisher’s exact test to account for frequency values < 5. The differences were considered significant at p < 0.05.

## Results

### Expressions of SATB1 in 29 SCLC patients

**Figure 1:**
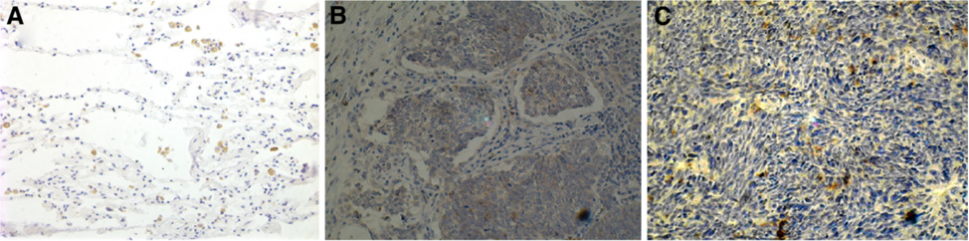
**Expression of SATB1 in 29 SCLC patients, positive expression of SATB1 located in the nucleus: A, in lung cancer adjacent tissue(X100); B, in SCLC tissue (X100); C, in metastasis lymphoid node (X100).**

As we can see in Figure 1 and Table 1, most positive SATB1 located in the nucleus. The expression of SATB1 in SCLC and metastatic lymph nodes tissues were significantly stronger than that of lung cancer adjacent tissue (p < 0.01). The expression of SATB1 in metastatic lymph nodes was slightly higher than that of SCLC tissues, but there were no significant difference (p > 0.05).

**Table 1:** Clinical characteristic of patients to the expression of MEKK1

**Variables**	**Patient number (n)**	**Staining score of SATB1**				**P**
		**0**	**1**	**2**	**3**	
Tissue						<0.001
Cancer	29	0	2	4	23	
Adjacent tissue	29	10	7	7	5	
Lymph node						0.141
Involved	11	0	1	1	9	
Free	18	0	1	2	15	

### SATB1-siRNA inhibited SATB1 expression in lung cancer cells

**Figure 2:**
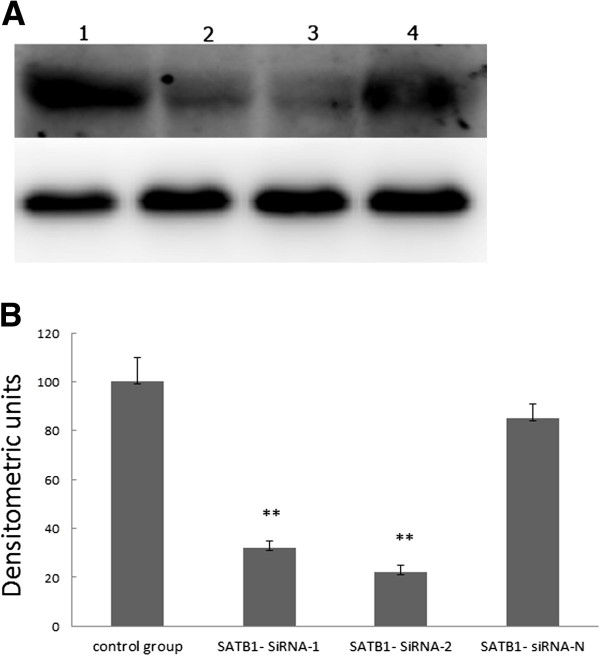
**SATB1-siRNA inhibited SATB1 expression in NCI-H446 cells. A**, Western blot of SATB1 in four groups: 1, control group; 2, transfected with SATB1- SiRNA-1; 3, transfected with SATB1- SiRNA-2; 4, transfected with SATB1- siRNA-N; **B**. Densitometric analysis of SATB1 protein levels.

As shown in Figure 2, for cells transfected with SATB1-siRNA-1 or SATB1-siRNA-2, a very low SATB1 expression was detected compared with that in control or SATB1-siRNA-N transfected cells.

### SATB1-siRNA induced morphology changes

**Figure 3:**
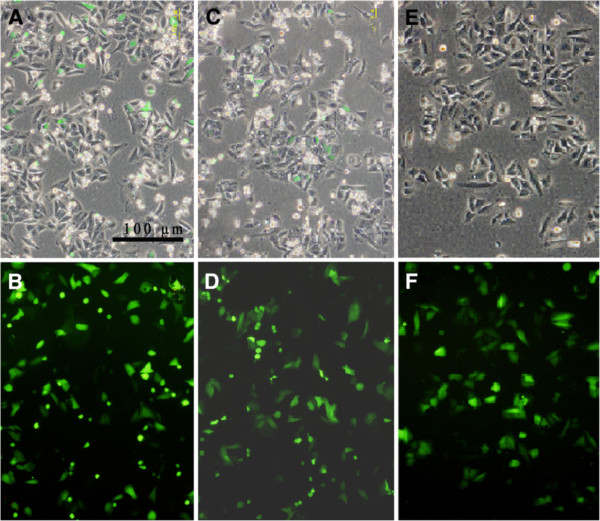
**SATB1-siRNA induced morphology changes of NCI-H446 cells 48 hours after transfected: A, B,transfected with SATB1- SiRNA-1(X100); C, D: transfected with SATB1- SiRNA-2(X100); E, F: transfected with SATB1- siRNA-N (X100).**

After transfected, green fluorescence could be observed in the cytoplasm of NCI-H446 cells. The cells transfected with SATB1- siRNA were less confluent, smaller and more rounded than that of control. Consistently, there were fewer cells in the view of fluorescence microscopy in SATB1-siRNA-1 and SATB1-siRNA-2 groups compared with SATB1-siRNA-N and control group cells 48 hours after transfection (Figure 3). The results imply that SATB1 participate in cell cycle progression and cell survival.

### SATB1-siRNA inhibited NCI-H446 cell proliferation and induced NCI-H446 cells apoptosis

**Figure 4:**
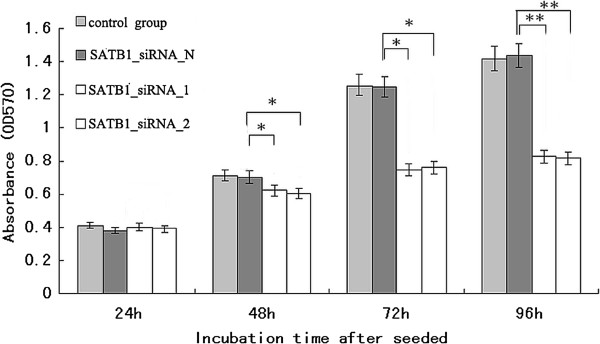
**SATB1-siRNA inhibited NCI-H446 cell proliferation.** *, P < 0.05; **, P < 0.01; compared with SATB1-SiRNA-N.

MTT analysis result showed (Figure 4) the proliferation of NCI-H446 cell was significantly inhibited by SATB1-siRNA-1 and SATB1-siRNA-2, but not by SATB1-siRNA-N, there was no significant difference between control group cell andSATB1-siRNA-N group cells.

**Figure 5:**
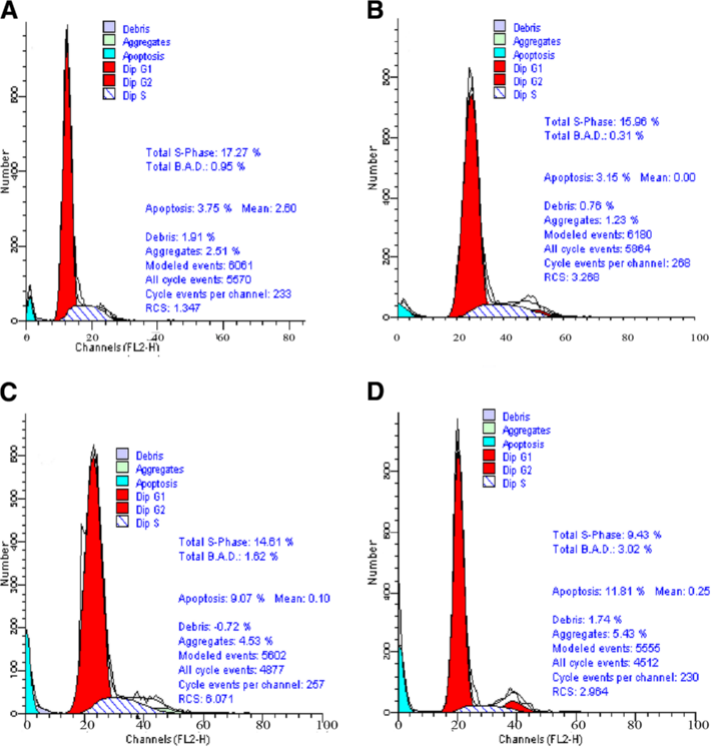
SATB1- siRNA induced NCI-H446 cells apoptosis after transfected with SATB1- SiRNA-1, SATB1- SiRNA-2, SATB1- siRNA-N, result showed by absorption value of PI stain at 24–96 hours.

The significant decrease in cell proliferation suggested that NCI-H446 cells treated with SATB1- siRNA might have underwent apoptosis. The propidium Iodide (PI) stain cells were analyzed by fluorescence activated cell sorting assay; result showed the apoptosis rates in SATB1-siRNA-1 and SATB1-siRNA-2 transfected cells were significantly higher than those in control and SATB1-siRNA-N transfected cells (Figure 5).

### SATB1-siRNA inhibited NCI-H446 cell invasion

**Figure 6:**
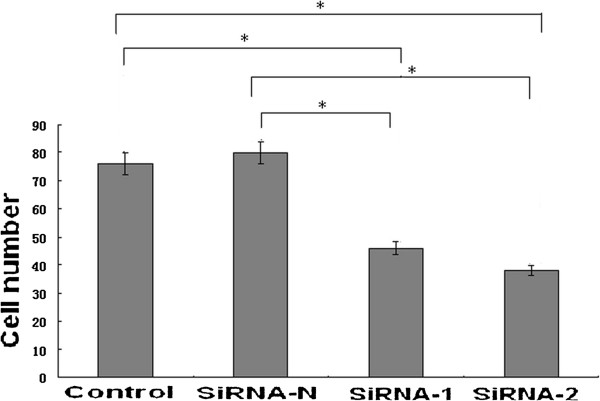
SATB1-siRNA inhibited NCI-H446 cell invasion, *, P < 0.05.

After NCI-H446 cell transfected, HE stain was conducted on the Matrigel-coated membranes. Result showed the cell number on the membrane was significantly decreased by interfering of SATB1-siRNA-1 and SATB1-siRNA-2 (Figure 6), and there was no significant difference between the siRNA-N group and control group (P > 0.05).

## Discussion

SCLC, of which lymph nodes metastasis usually occurs in early stage, is a special kind of lung cancers. Although radiotherapy and chemotherapy have produced modest benefits in some patients, it would relapse and resistant to many drugs after traditional therapies. This is particularly important for early detection and providing reliable therapeutic target.

In this study, we verified SATB1 was overexpressed in the SCLC tissues and metastatic lymph nodes tissues, small interfering RNA targeting SATB1- SATB1- siRNA-1 and SATB1-siRNA-2 were constructed successfully, our results showed that transfection with SATB1- siRNA-1 or SATB1-siRNA-2 into the SCLC cell-NCI-H446, could inhibit the cells proliferation and invasion significantly. In addition, SATB1-SiRNA could induce the apoptosis of SCLC cells in vitro.

SATB1 was originally characterized as a regulator in T cell differentiation, found to be overexpressed in metastatic breast cancer cell lines and in human tissue specimens from advanced stages of breast carcinomas with metastasis [2,4,19-21]. There are a few studies reported the expression of SATB1 in lung cancers, but the role of SATB1 is controversial: a study in squamous cell lung cancer and non-small cell lung cancers (NSCLC) showed that SATB1 expression was lost and the loss of SATB1 predicted poor prognosis in squamous cell carcinomas [22], Zhou et al. showed the expression of SATB1 mRNA was much higher in NSCLC tissues with or without metastasis than in normal lung tissues [23]. In our study, we found SATB1 was highly expressed in SCLC tissues and metastatic lymphoid nodes tissues compared with lung cancer adjacent tissue, SATB1-siRNA could effectively inhibit SATB1 expression in SCLC cells. Treatment of SCLC cell lines with SATB1-siRNA resulted in morphologic changes in these cells. Our result also showed SATB1-siRNA could induce SCLC cells apoptosis after transfecting SATB1-SiRNA into SCLC cells. These results suggest SATB1 might be an ideal target for the treatment of SCLC. Metastasis is the final step in solid tumor progression and is the most common cause of death in cancer patients. Controlling the invasion and migration can improve the survival rate of cancer patients. Recent study demonstrated that the SATB1 plays an important role in the process of invasion and migration. Consistent with the previous study, our results also showed the invasion ability of SCLC cells declined obviously after transfected with SATB1-SiRNA.

About the mechanisms why SATB1 influence the proliferation and invasion for small lung cancer cell, so far there is no much research on that, however, lots of study about the roles of SATB1 in breast cancer and other cancers have been investigated, Han et al. use gene chip to find SATB1 can induce the change of more than 1000 genes’ expression [3]. Some are associated with cancer invasion and metastasis, such as MMP2, MMP9, CTGF, et al. [6]. We will further study its detailed mechanism in the small lung cancer cell.

In summary, our work provides a better understanding of the physiological role of SATB1 in SCLC, SATB1 could regulate the invasion and migration of SCLC cells, this may provide important clues for more effective targeting of SCLC and other cancers with aberrant SATB1 activation.

## Competing interests

The authors declare no conflict of interests.
